# Bell’s palsy and lip HSV-1 infection: importance of subcutaneous access

**Published:** 2022-07-09

**Authors:** Marina S Boukhvalova, Emma Mortensen, Diego Lopez, Betsy C Herold, Jorge CG Blanco

**Affiliations:** 1 Sigmovir Biosystems, Inc., Rockville, MD, USA; 2 Albert Einstein College of Medicine, Bronx, NY, USA

**Keywords:** facial paralysis, bell’s palsy, HSV-1, demyelination, subcutaneous, cotton rat

## Abstract

Although HSV-1 has been implicated in facial palsy for a long time, testing and treating for HSV is not routine. The lack of a meaningful demonstration of how HSV-1 would cause facial palsy has limited progress in this field. Herein we demonstrate that the depth of the lip HSV-1 infection defines the course of the disease, with deeper subcutaneous infection allowing virus access to the facial nerve and causing facial palsy. HSV-1 inoculated subcutaneously caused extensive facial paralysis in cotton rats *Sigmodon hispidus*, while virus inoculated in the same area of the lip by skin surface abrasion did not. Demyelination along the facial nerve (CN VII) accompanied subcutaneous HSV-1 infection and was identified as the possible underlying mechanism of the disease. This causality demonstration is particularly important in light of increased facial palsy outbreaks associated with SARS-CoV-2 infection and SARS-CoV-2 and influenza vaccinations.

## Introduction

Cases where the etiology of acute unilateral facial paralysis cannot be established are called Bell’s palsy [1]. A hypothesis put forward in 1972 suggested that HSV could be responsible for a large percentage of cases of Bell’s palsy [2]. About a third of Bell’s palsy cases are currently thought to be attributable to HSV-1 [3]. However, the role of HSV-1 in causing facial palsy has not been universally accepted because connection between HSV-1 infection in the lip (the most common route) and facial palsy has never been conclusively demonstrated. Murine and Wistar rat models have been used to show that HSV-1 inoculation of the ear auricle can lead to facial nerve paralysis [4–6]. Auricular infection, however, is not a common route of HSV-1 acquisition in humans and the question persists as to how and if HSV-1 can cause facial nerve paralysis under more common conditions, such as the HSV-1 infection of the lip. As a result, routine testing for HSV is not commonly performed when patients present with facial palsy, the optimal sample to assess for HSV and sensitivity and specificity of assays are not known, and the best treatment has not been identified.

We recently demonstrated that lip HSV-1 infection of cotton rats, if administered by scratching of the lip surface (abrasion), results in virus entering the central nervous system (CNS) via trigeminal pathways and the development of symptoms associated with the brainstem/cerebellar damage (e.g., head tilt, compromised balance) and multifocal demyelination in the brainstem, cerebellum, and forebrain [7]. Surprisingly, in subsequent studies, we observed that infection with the same amount of virus in the same area of the lip, but delivered deeper (subcutaneously) instead of by superficial abrasion, resulted in findings resembling Bell’s palsy.

## Materials and methods

### Cells and virus:

HSV-1 (strain 17) (kindly provided by Priscilla Shaffer of Harvard Medical) was grown on Vero cells and stored at −80° C at a concentration of ∼10^8^ PFU/ml. Virus was diluted in PBS, pH 7.4, to appropriate concentration within an hour of infection and maintained on ice.

### Animals:

Inbred *Sigmodon hispidus* (*S. hispidus*) cotton rats were obtained from a colony maintained at Sigmovir Biosystems, Inc. Four to five week old animals of both sexes were used for the studies. Animals were housed in large polycarbonate cages and were fed a standard diet of rodent chow and water. The colony was monitored for antibodies to adventitious respiratory viruses and other common rodent pathogens and no such antibodies were found. All studies were conducted under applicable laws and guidelines and after approval from the Sigmovir Biosystems, Inc.’s Institutional Animal Care and Use Committee (IACUC).

### Animal Studies:

Cotton rats were anesthetized with ketamine/xylazine by injecting intramuscularly (i.m.) a mixture of 50 mg ketamine and 3.3 mg xylazine per 100 g body weight. Using a 300 μL syringe, fifty (50) μL of HSV-1 solution was injected subcutaneously in the right lip, upper vermilion border. Control animals were infected with the same amount of HSV-1 inoculated in the same area of the lip by abrasion: a 20 μl drop of virus was placed on the vermilion border of the upper right lip, followed by repeat abrasion of the area using a sharp needle. At various times after infection, animals were examined for the following symptoms: head tilt, loss of blink reflex, whisker touch response, and facial asymmetry. Blink reflex was evaluated by blowing air from a syringe into the right and left eye and recording successful or unsuccessful blinking on each side. Whisker touch response was evaluated by touching whiskers on the right and left side with a cotton tip applicator and monitoring twitching. Blink reflex and whisker touch response were evaluated three times on each side, head tilt and facial asymmetry were examined visually. Onset and recovery from symptoms was recorded. Saliva samples were collected using cotton swab pre-wet with PBS. The tip of the swab was placed in a tube with 200 μl PBS and flash-frozen in liquid nitrogen, prior to transfer to −80°C storage for subsequent DNA extraction. At various times after infection, animals were sacrificed by CO_2_ asphyxiation and samples were collected for virus quantification and/or gene expression analysis. The right trigeminal ganglion, the right geniculate ganglion, and the brain segments (forebrain, cerebellum, and brainstem) were collected and processed for RNA extraction and reverse transcription as described [20]. Brains for histopathology analysis were immersed in 10% buffered formalin and sectioned as described previously [7]. H&E-stained coronal segments corresponding to BST/pons and cerebellum showed the most pronounced changes. The presence of demyelinated lesions was documented and microphotographed.

### Virus titrations and PCR:

TG and GG homogenates were assayed for the presence of infectious HSV-1 by titering homogenates on Vero cells by plaque assay as described earlier [7]. For quantification of HSV-1 by PCR, DNA was extracted from saliva samples and HSV-1 polymerase level was quantified by qPCR using the following primers: forward: 5’ AGAGGGACATCCAGGACTTTGT 3’; reverse: 5’ CAGGCGCTTGTTGGTGTAC 3’. The same primers were used to analyze expression of HSV-1 mRNA in RNA prepared from TG, GG, and BST using methods described previously [8]. Expression of latent transcript was quantified based on the method of Gussow et al. 2006 [9]. In brief, latent transcript expression was defined as the ratio of spliced LAT (b and b’) (with primers detecting the transcript after the intron is spliced out) to 5’ LAT exon (a and a’) (with primers detecting either unspliced primary transcript or the spliced transcript) [9]. Spliced LAT was quantified using the following primers [9]: forward: 5’ CAACAAAGACGCCGCGTTT 3’; reverse: 5’ CCGCTTCCGCCTCCTC 3’; 5’ LAT exon was quantified using the following primers: forward: 5’ GGCTCCATCGCCTTTCCT 3’; reverse: 5’ AAGGGAGGGAGGAGGGTACTG 3’. Expression of myelin basic protein (MBP) in forebrain, cerebellum, and brainstem samples was quantified using the following primers: forward: 5’ ACTTCTTCAAGAACATTGTGAC 3’; reverse: 5’ CCCTTGAATCCCTTGTGAGCCG 3’.

### Statistical analyses:

Comparisons were performed by Student *t* test or ANOVA followed by Tukey *post hoc* test.

## Results

**Figure 1.**
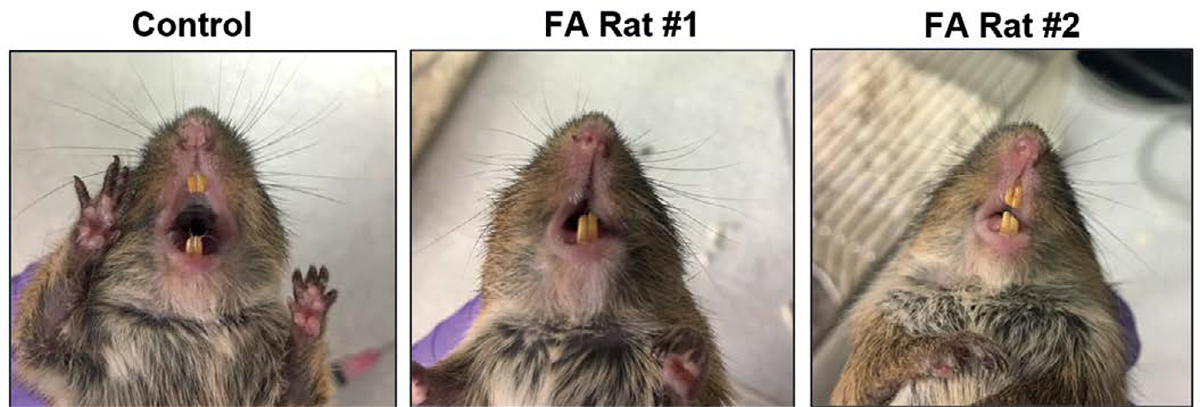
Facial palsy in cotton rats infected with HSV-1 in the lip subcutaneously. Faces of a control (uninfected) and two HSV-1-infected cotton rats with facial asymmetry (FA). Animals were infected with 10^5^ PFU HSV-1 subcutaneously (s.c.) in the right upper lip and photographed on day 6 post-infection (p.i.). Significant deformity of the face was seen in the infected rats, with elongated right upper lip, drooping right side of the face, and nose turned to the left.

Cotton rats *S. hispidus* inoculated with HSV-1 in the upper vermilion border of the lip subcutaneously developed facial asymmetry, defective blink and defective whisker touch response within a week of infection (Figure 1, Table 1). The findings were similar in male and female rats, and were transient with all animals recovering by days 17–19. All defects affected the right side of the face, ipsilateral to the site of infection, and several animals also had dry eyes on the ipsilateral side of infection (data not shown), concurrent with other symptoms. Spontaneous recurrence of facial palsy was detected in one animal. Facial palsy recurred in that animal about 3 weeks after the resolution of the first episode and spontaneously resolved within a week. Samples were not available to determine if the recurrence was associated with HSV-1 reactivation.

**Figure 2.**
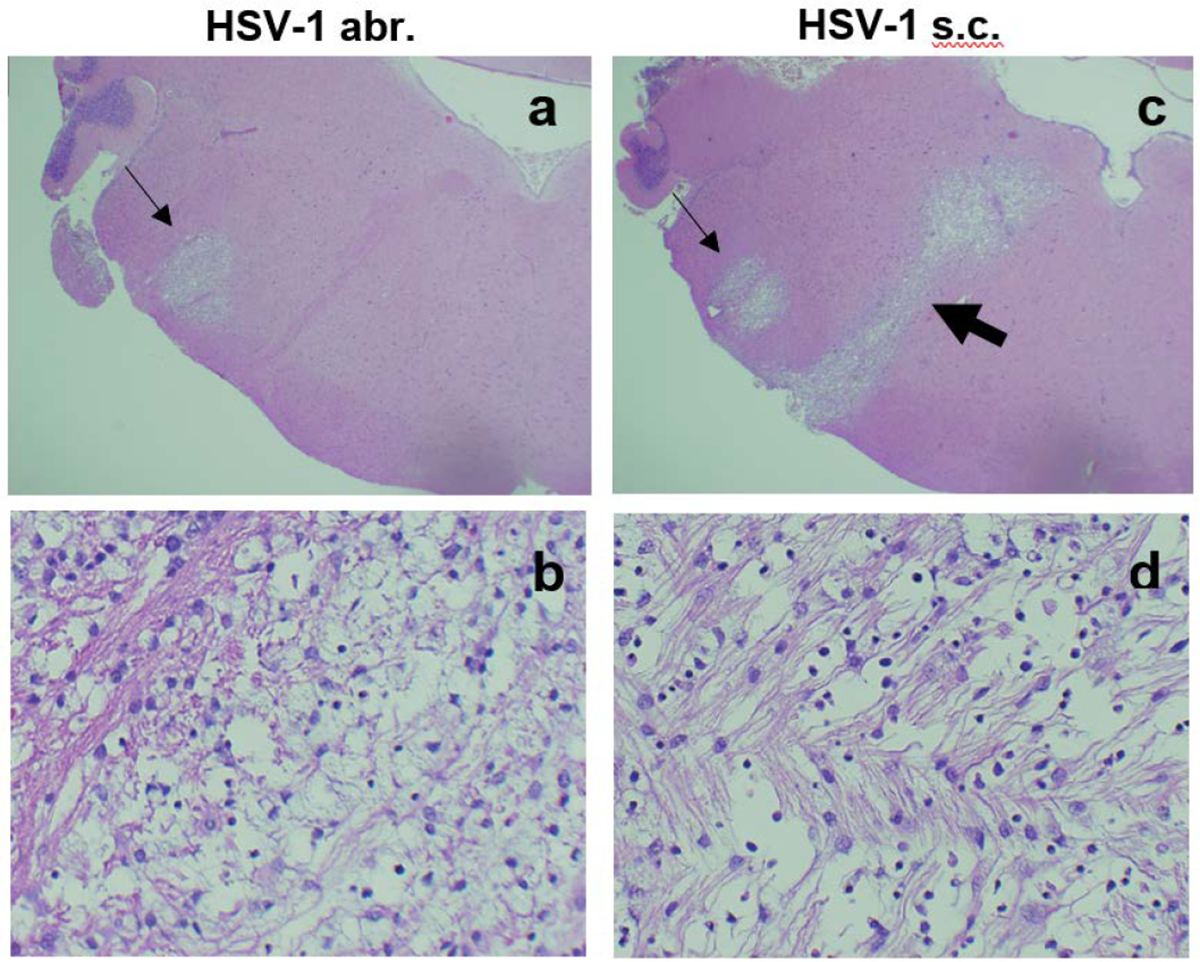
Brain pathology in cotton rats infected with HSV-1 in the lip subcutaneously (s.c.) or by abrasion (abr.). Brainstem (BST) of animals infected with 10^5^ PFU HSV-1 by abr. (a,b) or s.c. (c,d). (a,c) Lesions in the sensory trigeminal (ST) area corresponding to the trigeminal (CN V) nerve nuclei and tracts (thin arrows) could be seen in animals infected either by abr. (a) or s.c. (c). Lesions in the facial nerve (CN VII) area (thick arrow) were seen only animals infected s.c. (c). H&E stain, 20X. (b,d) Different organization of fibers within the ST (b) and CN VII (d) lesions. H&E stain, 400X.

**Figure 3.**
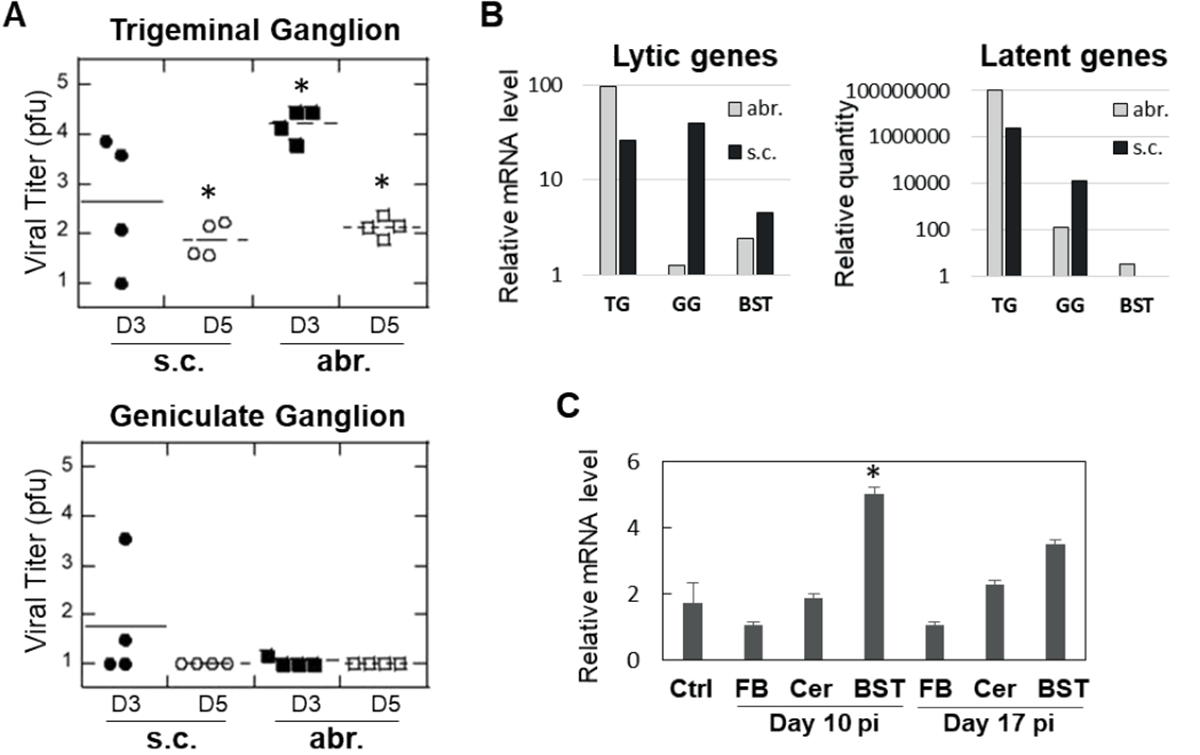
Detection of HSV-1 in trigeminal (TG) and geniculate (GG) ganglia and changes in myelin basic protein (MBP) expression. (A) TG and GG were harvested from HSV-1-infected animals on days 3 and 5 post-infection (D3 and D5, respectively). HSV-1 viral load was quantified by plaque assay on Vero cells. *p<0.05 compared to uninfected controls. (B) Expression of lytic (HSV-1 polymerase) or latent (spliced LAT) transcripts was analyzed by quantitative PCR in TG, GG, or brainstem (BST) in animals sacrificed 14 days after infection. A pool of samples from 3 animals per abr. or s.c. group was used, with all reactions run in duplicates. (C) Expression of MBP mRNA is increased at the time of maximum remyelination in the brainstem (BST). Animals were infected subcutaneously with HSV-1 and the forebrain (FB), cerebellum (Cer), and BST were collected on days 10 and 17 post-infection (pi) for MBP gene expression analysis by qPCR. *p<0.05 compared to uninfected controls (Ctrl).

Comparison of brain histopathology of animals infected subcutaneously or by abrasion showed clear differences. Animals infected by abrasion had large lesions in the sensory trigeminal (ST) area (corresponding to the trigeminal nerve nuclei and tracts), while animals infected subcutaneously had smaller lesions in the ST area, but more pronounced demyelinated lesions in the facial nerve (Figure 2). Although only hematoxylin and eosin (H&E) images are presented here, the previous work showed that H&E demonstrates demyelination comparable to what is observed with Luxol Fast Blue stain [7]. The lesions were also found in the cerebellum of abrasion-infected animals, but not in the cerebellum of subcutaneously-infected animals (Table 2). These results indicate that different neuroanatomical structures are affected when HSV-1 is administered subcutaneously versus by abrasion and that facial nerve demyelination is specific to subcutaneous infection. The limitations of the current technique (one section per brainstem sample) did not allow us to visualize all planes in which demyelinated facial nerves could be located, but it is anticipated that the effect may be more widely represented than shown in Figure 2. Analysis of TG and GG viral load after infection showed important differences between the two routes of infection: more HSV-1 was detected in the TG of animals infected by abrasion, while more virus was detected in GG after subcutaneous infection (Figure 3A). A higher expression of HSV-1 lytic and latent transcripts was detected in GG of animals infected subcutaneously compared to animals infected by abrasion (Figure 3B). Detection of HSV-1 latent transcript in GG is consistent with previous clinical studies [10]. HSV-1 was also detected in saliva of cotton rats with facial palsy (data not shown), similar to what has been described for symptomatic Bell’s palsy patients [3,11–13]. Increased expression of myelin basic protein (MBP) (involved in the process of remyelination) was detected in the BST of HSV-1-infected cotton rats on day 10 post-infection and subsided by day 17 (Figure 3C), the time by which most animals recovered from facial palsy, suggesting that remyelination may aid recovery from facial palsy.

## Discussion

**Figure 4.**
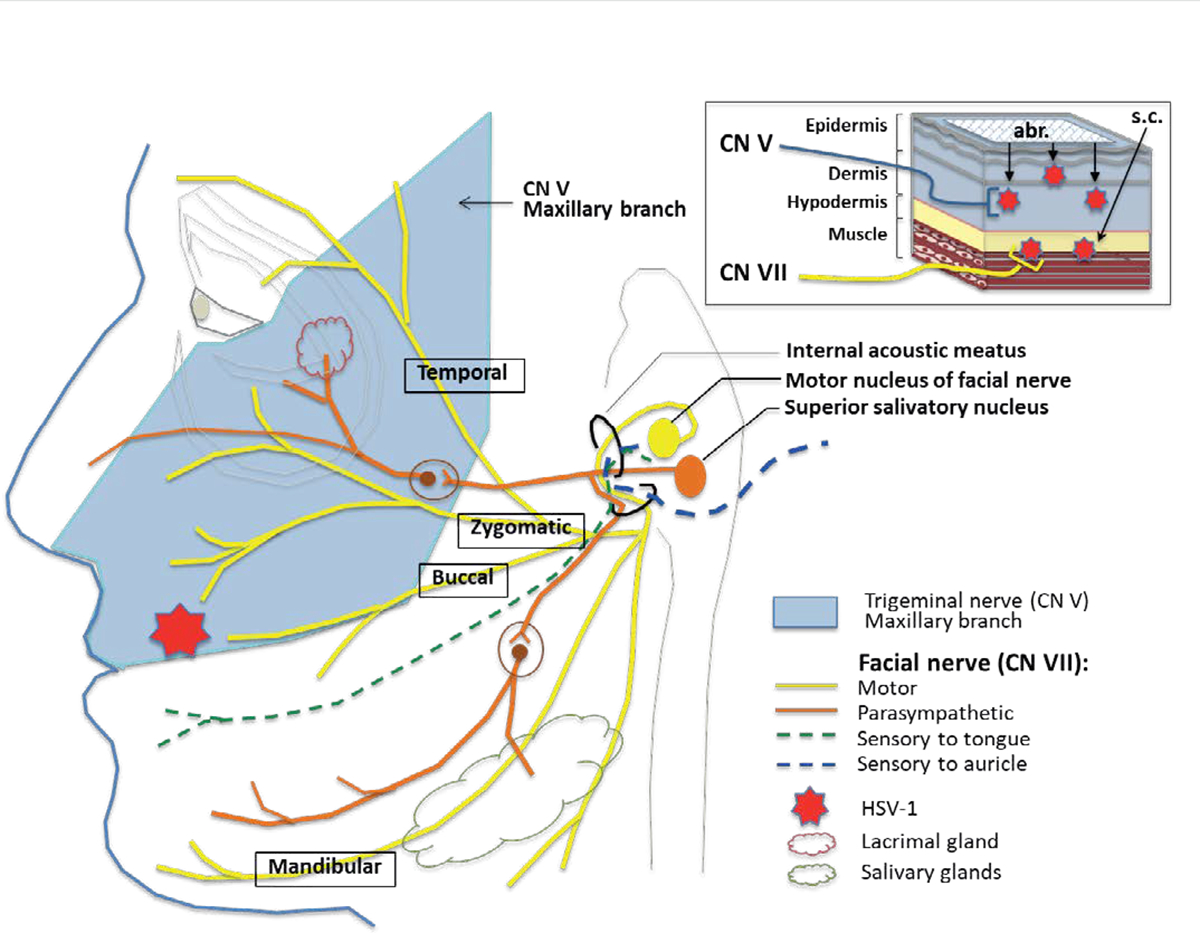
Proposed mechanism of facial palsy induction by deep lip HSV-1 infection. Facial palsy arises when HSV-1 gains access to subcutaneous (s.c.) tissues of the lip through a deep skin cut and virus reaches motor nerve endings of facial nerve CN VII (upper right diagram). In contrast to subcutaneous infection, superficial infection in the same area of the lip by abrasion (abr.) leads to HSV-1 being picked up by the more superficially located sensory nerve endings of trigeminal nerve CN V (maxillary branch) innervating the same area of the lip.

These results overall demonstrate that acute lip HSV-1 infection can cause facial palsy if virus gains access to subcutaneous tissues (e.g., through a deep skin cut) and that demyelination along the facial nerve due to HSV-1 infection (and associated inflammation) could be the underlying cause of the disease. This hypothesis is consistent with the superficial location of sensory nerve endings of CN V and deeper location of the motor nerve endings of CN VII in the same area of the lip (Figure 4) and our findings of demyelination along the CN V or CN VII pathways in the brain. It is also consistent with the signs of lip HSV-1 disease in cotton rats, where infection via abrasion causes symptoms linked to brainstem/cerebellum involvement (e.g., head tilt, compromised balance) while subcutaneous infection leads to facial palsy (e.g., sagging mouth, defective blink response) with involvement of both lower and upper parts of the same side of the face. The defective blink response developing shortly after subcutaneous HSV-1 infection and dry eye on the side of infection suggest that shortly after entering the facial nerve through its buccal or zygomatic branches in the lip, HSV-1 can access other CN VII branches, including the temporal branch involved in the blink reflex and its parasympathetic branches innervating lacrimal, submandibular, and sublingual glands (Figure 4). This hypothesis is consistent with the detection of HSV-1 in saliva samples and development of eye symptoms. It is also consistent with the fact that demyelination in cotton rats coincides with the onset of symptoms of facial palsy, and the recovery coincides with the remyelination along the facial nerve in the BST.

Demonstration that this mode of HSV-1 infection can lead to facial palsy is an important step toward better understanding of the facial nerve paralysis as it addresses a novel mechanism of the disease and facial nerve paralysis. Younger cotton rats in our studies were more likely to develop facial palsy upon subcutaneous infection than older animals (data not shown). This suggests that there is a narrow window in neurologic development when HSV-1 propensity for causing facial palsy might be high. Acute infection with HSV-1 through the deep cut in the lip thus is likely to occur in naïve human adolescents or young adults during kissing/contact with an HSV-1-positive individual who is shedding virus at that time. On the days when viral lesions are present, more than 8 log_10_ copies/ml of HSV-1 can be detected throughout oral mucosa of infected individuals [14]. Therefore, the combination of a deep cut and an initial high viral dose of HSV-1 is achievable in nature and our studies appear to model it. Once infected by this mode, an individual would harbor virus in geniculate ganglia and can reactivate and have repeat episodes under conditions of stress or other factors. Importantly, the model can be used for testing novel therapeutic interventions against Bell’s palsy. The currently recommended approach for treatment of Bell’s palsy includes a combination of corticosteroids and antivirals [15]. Incomplete recovery, however, is still seen in some cases and may negatively affect the quality of life [16]. Because demyelination was identified as one of the main characteristics of the disease, and since demyelination occurs at the time patients are likely to seek medical help, promoting remyelination may be the method to ensure faster and more complete recovery from the disease.

The model can also be used to assess possible association between certain respiratory viral infections, vaccines, and Bell’s palsy. Inactivated intranasal influenza vaccine used in Switzerland in 2000–2001 increased incidence of Bell’s palsy 19-fold and was withdrawn from use [17]. While parenteral inactivated influenza vaccines available in the United States are generally very safe, there have been isolated reports of HSV reactivation (both HSV-1 and HSV-2) in individuals receiving influenza vaccination [18–19]. Higher occurrence of facial palsy during COVID-19 outbreak of SARS-CoV-2 has been noted [20]. It could be due to a direct neuropathic effect of SARS-CoV-2 (or its individual proteins), or an indirect effect due to HSV-1 reactivation under the highly stressful environment of the COVID pandemic. Cases of Bell’s palsy have also been reported during mRNA SARS-CoV-2 vaccine trials [21]. While these cases were few and did not appear to exceed the rate expected in the general population, it would still be important to assess the interplay between SARS-CoV-2, neuropathy, stress, HSV-1 infection/reactivation, and facial palsy.

## Figures and Tables

**Table 1.** A summary of facial asymmetry (FA, shaded in pink) and other findings of disease that developed in HSV-1-infected animals as a function of time (day p.i.). Four- to-five-week-old animals were infected with 10^5^ PFU HSV-1 in the lip s.c. and monitored for 40 days after infection. FA was often accompanied by additional symptoms, including defective right eye blink response (RB) and defective right whisker touch response (RT). One animal was found dead on day 10 p.i. (shaded cells). One animal had a recurrence of symptoms between days 33 and 38 p.i.

	Animal #	Day post-infection															
		D0	D4	D5	D6	D7	D10	D11	D12	D13	D15	D17	D19	D28	D33	D38	D40
**Fem** **s.c.**	119893				RB, RT, FA	RB, RT, FA	RB, RT, FA	RB, RT, FA	RT, FA	RT, FA	RT, FA						
	119894				FA	RT, FA	RT, FA	RT, FA	RT, FA	RT, FA							
	119895				FA	RB, RT, FA	RB, RT, FA	RB, RT, FA	RB, RT, FA	RB, RT, FA	RT, FA						
	119896				RB, RT, FA	RB, RT, FA	RB, RT, FA	RB, RT, FA	RT, FA	RT, FA	RT, FA						
	119897				RB, RT, FA	RB, RT, FA	RB, RT, FA	RT, FA	RT, FA	RT, FA	RT, FA						
	119898				RB, RT, FA	RB, RT, FA	RB, RT, FA	RT, FA	RT, FA	RT, FA	RT, FA						
	119899				RB, RT, FA	RB, RT, FA	RB, RT, FA	RB, RT, FA	RB, RT, FA	RB, RT, FA	RT, FA						
	119900				RB, RT, FA	RB, RT, FA	RB, RT, FA	RB, RT, FA	RB, RT, FA	RB, RT, FA	RT, FA						
	119902				RB, RT, FA	RB, RT, FA	RB, RT, FA	RB, RT, FA	RB, RT, FA	RT, FA	RT, FA						
	119903				FA	RB, RT, FA	RB, RT, FA	RT, FA	RT, FA	RT, FA	RT, FA						
**Male** **s.c.**	119905			RB	RB, RT, FA	RB, RT, FA	RB, RT, FA	RT, FA	FA	RT, FA	RT, FA						
	119906							FA	FA								
	119907						RT, FA	RT, FA	RT, FA	RT, FA	FA						
	119908					RB, RT, FA	RT, FA	RB, RT, FA	RT, FA	RT, FA	RT, FA						
	119909				RB, RT, FA	RB, RT, FA	RB, RT, FA	RB, RT, FA	RT, FA	RB, RT, FA	RT, FA						
	119910				FA	RB, RT, FA	RB, RT, FA	RB, RT, FA	FA	RT, FA	RT, FA						
	119911				RB, RT, FA	RB, RT, FA											
	119912				FA	RB, RT, FA	RB, RT, FA	RB, RT, FA	RT, FA	RT, FA	FA	FA					
	119913					RB, RT, FA	RB, RT, FA	RB, RT, FA	RB, RT, FA	RB, RT, FA	RT, FA				RB, RT, FA	FA	

**Table 2.** Summary of histopathologic changes in the BST/pons and cerebellum of female (Fern) and male (Male) cotton rats infected with HSV-1 by abr. or s.c. and sacrificed on days 10–11 post-infection

	Animal #	BST/Pons				Cerebellum	
		ST(CN V) lesions	Inflam.	CN VII lesions		lesions	total # of lesions
**Fem** **abr.**	119845	+	+	−		+	4
	119847	+	+	−		+	3
	119849	+	++	−		+	2
	119850	+	−	−		−	−
**Male** **abr.**	119859	+	−	−		+	1
	119862	+	+	−		+	2
	119864	+	−	−		+	3

**Fem** **s.c.**	119901	+	+	+		−	−
	119904	+	+	−		−	−
	119918	+	+	−		−	−
	119927	−	++	+		−	−
**Male** **s.c.**	119915	+	−	−		−	−
	119916	−	++	+		−	−
	119938	−	++	−		−	−
	119939	+	++	+		−	−
	119940	−	+++	−		−	−

## Abbreviations:

HSV-1: Herpes Simplex Virus 1
